# Взаимодействие микробиома кишечника и пероральных сахароснижающих препаратов: обзор литератур

**DOI:** 10.14341/probl12835

**Published:** 2022-04-30

**Authors:** А. В. Буйваленко, Е. В. Покровская

**Affiliations:** Национальный медицинский исследовательский центр эндокринологии; Национальный медицинский исследовательский центр эндокринологии

**Keywords:** микробиом, микробиота, сахароснижающие препараты, метформин, лекарственное воздействие

## Abstract

Кишечная микробиота представляет собой совокупность микроорганизмов, населяющих кишечник человека. Основными функциями кишечной микробиоты являются: получение энергии из сложных волокон пищи, созревание и образование иммунной системы, кишечный ангиогенез, восстановление эпителиального повреждения кишечника, развитие нервной системы, защита от патогенов и т.д. Взаимодействие между микробиотой кишечника и лекарственными препаратами, кроме антибактериальных, является сложным и двунаправленным: медикаменты влияют на разнообразие микроорганизмов, и, наоборот, микробиота кишечника усиливает реакцию организма на лекарственное вещество путем ферментативного преобразования структуры молекулы, изменения ее биодоступности, биологической активности или токсичности. Исследование взаимоотношений между лекарственными средствами, микробиотой и хозяином является сложной задачей, а биологические механизмы, лежащие в основе этих взаимодействий, еще недостаточно изучены. В этом обзоре мы обсуждаем двунаправленное взаимодействие между микробиомом кишечника человека и сахароснижающими препаратами, описываем изменения состава микробиоты при их применении, а также оцениваем потенциальные клинические последствия для организма человека. Получение знаний в этой области помогут проложить путь к разработке новых стратегий, основанных на принципах жизнедеятельности микробиоты, которые в будущем можно будет использовать для улучшения результатов лечения сахарного диабета 2 типа (СД2).

## Abstract (en)

The gut microbiome is the largest microbial habitat in the human body. The main functions include obtaining energy from complex food fibers, maturation and formation of the immune system, intestinal angiogenesis, restoration of epithelial damage to the intestine, development of the nervous system, protection against pathogens, etc. It is also known that a number of drugs can cause changes in the composition of the intestinal microflora, and intestinal bacteria, in turn, produce a number of enzymes and metabolites that can chemically change the structure of drugs, leading to more side effects, and in some cases to positive changes. In this review we present current evidence supporting the effects of microbiota in host-drug interactions, in particular, the reciprocal effects of gut microbiota and oral hypoglycemic drugs on each other. Gaining and evaluating knowledge in this area will help pave the way for the development of new microbiota-based strategies that can be used in the future to improve treatment outcomes for type 2 diabetes mellitus (T2D).

## МЕТОДОЛОГИЯ ПОИСКА ИСТОЧНИКОВ

Алгоритм поиска информации был разработан в соответствии с требованиями и положениями отчетности для литературных обзоров (PRISMA) в базе данных PubMed и GoogleScholar и включал поиск научной литературы и исследований с использованием поисковых запросов, ключевых слов (в т.ч. MeSH) и логических операторов. Двое из авторов независимо друг от друга изучили заголовки и аннотации публикаций на соответствие заявленной теме, возникшие разногласия решали путем переговоров. После анализа заголовков и их аннотаций непосредственно поставленной цели соответствовало 34 публикации. Последний поиск осуществлялся 14 августа 2021 г.

## ВВЕДЕНИЕ

Микробиота — это термин, который используется для характеристики микробиоценоза отдельных органов и систем [1]. Состав микроорганизмов различается в зависимости от локализации: кожа, кровь, ротовая и носовая полости и желудочно-кишечный тракт (ЖКТ) [2]. На микробиоту кишечника человека влияет множество факторов, таких как возраст, географическое положение, диетические особенности и вкусовые предпочтения, а также физическая нагрузка. Микробиота постоянно развивается, растет и адаптируется в зависимости от факторов окружающей среды и взаимоотношений бактерий. Видовое разнообразие бактерий в этих сообществах огромно, микроорганизмы взаимодействуют на различных уровнях посредством мутуалистических, комменсальных, конкурентных или иных отношений [3].

Всего несколько десятилетий назад оценить вклад микробиоты кишечника в здоровье человека технически было крайне затруднительно. Исследования микробиоты проводились с использованием методов культивирования, в которых выделялись один или несколько видов бактерий и изучались их свойства. Разработка метода секвенирования бактериального гена 16S рибосомальной РНК [4] позволила провести общую таксономическую оценку микробиома кишечника, что значительно расширило наши знания о широких вариациях в его микробном составе. В последнее время одним из главных методов изучения микробиома является полногеномное или метагеномное секвенирование (MGS). MGS позволяет идентифицировать не только бактерии, но и вирусы, а также простейшие и грибы, произвести анализ бактериальных генов и предсказать биологические пути их существования. Однако, как и в случае со всеми другими методами, основанными на секвенировании, результаты MGS зависят от метода, используемого для выделения ДНК из образцов стула, что является основной причиной технической вариабельности результатов исследований микробиома [5].

За последнее десятилетие сделано много интересных открытий, демонстрирующих связь состава микробиоты кишечника и возникновения различных заболеваний. Исследование ассоциаций микроорганизмов также показало корреляцию между видовым разнообразием и возникновением воспалительных заболеваний кишечника, синдромом «раздраженного кишечника», колоректального рака, а также заболеваний других органов и систем. Помимо исследования микробных ассоциаций, интервенционные исследования и исследования на животных подтверждают не только влияние микроорганизмов на определенные процессы, но и объясняют причинно-следственные связи между ними. Одно из недавних исследований показало, что многие используемые препараты, в частности, сахароснижающие средства, изменяют состав и функции микробиоты кишечника, в свою очередь, микробиота взаимодействует с веществами, попавшими в организм человека, в связи с чем, по всей видимости, влияние является двунаправленным [6].

Резюмируя вышесказанное, взаимодействие лекарственных препаратов с кишечной микробиотой может привести к уменьшению эффективности препарата по причине трансформации лекарственной молекулы бактериями и изменения ее биодоступности. Однако в зависимости от класса сахароснижающих препаратов эти изменения варьируют.

## Бигуаниды (метформин)

Метформин, относящийся к классу бигуанидов, является препаратом 1-й линии для лечения сахарного диабета (СД2). До недавнего времени исследования механизма действия метформина в основном были сосредоточены на его антиглюконеогенном действии на печень [7][8]. Однако в настоящее время появляется все больше сведений о том, что микробиота кишечника является ключевым медиатором терапевтического эффекта метформина (рис. 1). В соответствии с этой гипотезой внутривенное введение препарата для снижения уровня глюкозы в крови менее эффективно по сравнению с пероральным приемом [9][10].

**Figure:**
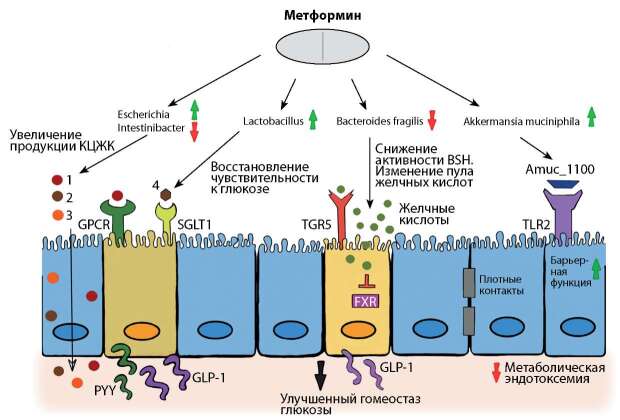
Рисунок 1. Метформин изменяет состав микробиоты кишечника, регулируя гомеостаз глюкозы1, 2, 3 — бутират, ацетат, пропионат соответственно; 4 — глюкоза; TGR5 — Takeda G protein–coupled receptor; BSH — bile salt hydrolase (гидролаза желчных кислот); FXR — фарнезоидный ядерный рецептор, чьим эндогенным лигандом являются желчные кислоты.Источник: Pryor R, Martinez-Martinez D, Quintaneiro L, Cabreiro F. The Role of the Microbiome in Drug Response. Annu Rev Pharmacol Toxicol. 2020 Jan 6;60:417-435. doi: 10.1146/annurev-pharmtox-010919-023612. Epub 2019 Aug 6. PMID: 31386593.Figure 1. Metformin alters the composition of the gut microbiota by regulating glucose homeostasis

В большинстве случаев метформин назначается при установленном СД2, что не позволяет разграничить воздействие препарата на микробиоту от изменений, которые связаны с основным заболеванием. В нескольких метагеномных исследованиях сообщалось, что люди с СД2 имеют измененный состав микробиоты кишечника по сравнению со здоровыми людьми [11], а именно, микробиота кишечника лиц, не принимающих метформин, характеризовалась истощением таксонов, продуцирующих бутират, включая виды Roseburia, Subdoligranulum spp., Clostridiales spp. Напротив, при приеме метформина увеличивалось количество Escherichia spp. и увеличивалась численность Intestinibacter spp. [11]. В одном из проведенных исследований было показано, что ранее наблюдаемые изменения в микробиоме кишечника, которые, как считалось, были вызваны наличием СД2, на самом деле обусловлены использованием метформина [11].

В другом исследовании сообщалось, что схожие изменения в микробиоте людей с СД2, ранее не получавших лечение, наблюдались после приема метформина в течение 4 мес, что позволяет предположить, что лечение действительно эффективно [12]. Было высказано предположение, что изменения в численности бактериальных таксонов могут опосредовать терапевтическое действие метформина за счет увеличения продукции короткоцепочечных жирных кислот (КЦЖК) [11][12], которые улучшают гомеостаз и метаболизм глюкозы на моделях грызунов [13]. Более того, известно, что до одной трети пациентов, принимающих метформин, сообщают о побочных эффектах со стороны желудочно-кишечного тракта (ЖКТ), таких как диарея, вздутие живота и тошнота, что можно связать с факторами вирулентности и генами газового обмена (в основном происходит из-за увеличения количества видов E. coli), которые могут являться причиной этих побочных эффектов [11]. Помимо вышеперечисленного, метформин нарушает метаболизм бактериального фолата у Caenorhabditis elegans [14], что может объяснить ухудшение фолатного статуса [15]. С другой стороны, вызванные метформином изменения микробиоты могут регулировать метаболизм глюкозы благодаря сохранению целостности кишечного барьера путем увеличения относительной численности A. muciniphila как у мышей [16], так и у людей [12].

Интересно, что белок внешней мембраны, выделенный из A. muciniphila, активирует толл-подобный рецептор 2-го типа (TLR2) и повторяет положительные эффекты интактного белка A. muciniphila на гомеостаз глюкозы и липидов у мышей [17]. Возможно, передача сигналов TLR2 может усиливать барьерную функцию кишечника и, следовательно, корректировать метаболическую эндотоксемию, связанную с СД.

В другом исследовании было высказано предположение, что микробиота кишечника опосредует эффекты метформина, влияя на секрецию гормонов кишечника. У лиц, принимающих метформин, обнаружено повышение уровня инкретинового гормона глюкагоноподобного пептида 1 (GLP-1) в плазме, а в некоторых исследованиях также сообщалось о сопутствующем повышении уровня пептида YY (PYY), который принимает участие в контроле аппетита [18]. Возможная связь между действием метформина на секрецию гормонов кишечника и микробиотой была впервые выявлена, когда наблюдалась корреляция между уровнями PYY и изменениями численности Bacteroidetes и Firmicutes spp. в образцах от пациентов с СД2, получавших монотерапию метформином [19]. Кроме того, есть доказательства того, что КЦЖК могут запускать секрецию GLP-1 и PYY энтероэндокринными клетками либо посредством взаимодействия с рецепторами, связанными с G-белком, либо посредством их ингибирующей активности гистондеацетилазы, либо же воздействуя на них как источник энергии [20]. Следовательно, метформин может косвенно стимулировать высвобождение этих гормонов, способствуя росту видов, продуцирующих КЦЖК.

Было обнаружено, что экспрессия молекулы натрий-глюкозного котранспортера-1 (SGLT-1), основного переносчика, ответственного за стимулированную глюкозой секрецию GLP-1, снижается в верхних отделах тонкой кишки крыс, получавших диету с высоким содержанием жиров [21]. Однако лечение метформином восстанавливало экспрессию SGLT-1 и чувствительность к глюкозе, а также увеличивало относительную численность Lactobacillus. Важно отметить, что трансплантация предварительно обработанной метформином микробиоты в тонкий кишечник крыс, получавших диету с высоким содержанием жиров, также восстанавливала экспрессию SGLT-1 и чувствительность к глюкозе, поддерживая механизм, опосредованный кишечной микробиотой. Требуются дальнейшие исследования, чтобы точно установить, как Lactobacillus активирует этот сенсор питательных веществ, чтобы усилить секрецию GLP-1 и снизить уровень глюкозы в плазме.

Взаимосвязь между метформином и микробиомом кишечника показывает, насколько молекула лекарственного средства может изменить микробиом кишечника, как влияет микробиота кишечника на действие метформина как лекарственной молекулы, а также объяснить возникновение побочных эффектов от препарата. Подобное влияние подчеркивает необходимость строгого контроля приема метформина, особенно при проведении исследований микробиома, направленных на изучение конкретных заболеваний или состояний.

## Агонисты ГПП-1

Имеется несколько сообщений о связи между агонистом рецепторов GLP-1 лираглутидом и измененной структурой микробиоты кишечника [22–25]. В целом было показано, что лечение лираглутидом снижает относительное количество связанных с ожирением бактериальных филотипов в моделях СД2 и ожирения на грызунах [22][24], и в одном из исследований наблюдаемое увеличение количества Lactobacillus отрицательно коррелировало с уровнем глюкозы в крови [23]. Повышение уровня A. muciniphila также наблюдалось в ответ на лечение лираглутидом у пациентов с СД2, что, возможно, отражает улучшение барьерной функции кишечника [25].

Кроме того, некоторые ингибиторы дипептидилпептидазы-4 (DPP-4), которые используются для повышения уровня GLP-1, также могут оказывать положительное воздействие через микробиоту кишечника. Например, лечение вилдаглиптином вызывало снижение продукции Oscillibacter и увеличение продукции как Lactobacillus, так и КЦЖК у мышей, получавших западную диету [26], а прием ситаглиптина также показал некоторые улучшения микробного профиля у лабораторных животных с индуцированным СД2.

## Акарбоза

Акарбоза — ингибитор α-глюкозидазы, который участвует в метаболизме глюкозы, задерживая переваривание сложных углеводов в тонком кишечнике. Поскольку этот препарат воздействует на субстрат, доступный для бактериальной ферментации, он может избирательно способствовать росту определенных таксонов и, таким образом, воздействовать на микробиоту. Действительно, несколько исследований продемонстрировали, что акарбоза изменяет состав бактериальных сообществ в кишечнике [27–29]. В рандомизированном контролируемом исследовании с участием 52 лиц с нарушением толерантности к глюкозе лечение акарбозой было связано со значительным увеличением количества Lactobacillus и Dialister spp., причем, число последних отрицательно коррелировало с уровнем глюкозы в крови [29]. Можно предположить, что изменения в структуре кишечной микробиоты могут вносить вклад в терапевтический эффект препарата. В другом исследовании обнаружена тесная связь между вызванными акарбозой изменениями микробиоты кишечника и количеством желчных кислот, которые были связаны с улучшением контроля гликемии [28].

## Тиазолидиндионы и препараты сульфонилмочевины

Лечение пиоглитазоном из класса тиазолидиндионов подавляло увеличение численности бактерий Proteobacteria, наблюдаемое у грызунов, получавших пищу с высоким содержанием жиров [30], в то время как лечение розиглитазоном восстанавливало пространственное распределение бактерий по слизистой оболочке подвздошной кишки, но не восстанавливало их состав [31]. Аналогичным образом исследование, изучающее влияние глипизида, препарата из группы сульфонилмочевины, на микробиоту людей с СД2, не показало значительных изменений в относительной численности как на уровне видов, так и на уровне микробиома. Однако было обнаружено, что биодоступность родственного препарата гликлазида повышается у диабетических крыс после введения пробиотиков, что подчеркивает возможные взаимодействия между лекарственными средствами и микробиотой [31].

## Ингибиторы SGLT-2 (глифлозины)

Ингибиторы SGLT-2 — одна из наиболее современных и перспективных групп сахароснижающих препаратов, реализуют свою функцию независимо от работы β-клеток островков Лангерганса поджелудочной железы. Глифлозины блокируют SGLT-2, локализованный в мембране эпителиоцитов проксимальных канальцев нефрона, тем самым препятствуя реабсорбции глюкозы [32]. В различных клинических исследованиях был отмечен широкий спектр метаболических эффектов ингибиторов SGLT-2, среди которых положительными считались снижение массы тела пациентов, уменьшение уровня триглицеридов в крови [33]. Вопрос влияния ингибиторов SGLT-2 на микробиом кишечника рассматривался в исследовании D.M. Lee и соавт., основная цель которого заключалась в оценке жесткости артериальной стенки мышей, получающих глифлозины в качестве противодиабетической терапии. Если на состояние сосудов ингибиторы SGLT-2 оказывали достоверный эффект, проявляющийся увеличением сосудистой эластичности на фоне гипонатриемии, то состав микробиома кишечника по сравнению с контрольной группой не продемонстрировал каких-либо значимых изменений [34]. Лечение дапаглифлозином, по-видимому, мало повлияло на микробиоту у контрольных мышей, но вызвало незначительные изменения в разнообразии микробных сообществ у животных с диабетом. В целом соотношение Firmicutes:Bacteroides было снижено у животных, получающих препарат, по сравнению с другими группами. При этом стоит отметить, что в группе приема препарата был отмечен рост Akkermansia muciniphila, что также сопровождалось снижением маркеров воспаления, массы тела и уровня глюкозы в крови.

## ОБСУЖДЕНИЕ И ВЫВОДЫ

Несмотря на обилие проведенных исследований и полученных выводов, на сегодняшний день из всех групп сахароснижающих препаратов наиболее изученным в отношении влияния на кишечную микробиоту является метформин. Результаты многих исследований являются несогласованными между собой, иногда с диаметрально противоположными исходами, что может быть обусловлено этническими особенностями, влиянием большого количества внешних факторов и разницей в дизайне исследований. Необходимы дальнейшие исследования для оценки существования причинно-следственной связи между изменениями микробиоты, вызванными сахароснижающими препаратами и их терапевтическими эффектами, на биологических моделях и на схожих по различным характеристикам выборках пациентов с учетом региона их проживания, пищевых привычек, приема других групп препаратов и прочих факторов, потенциально способных вносить вклад в изменения кишечной микробиоты. Четкое понимание механизмов взаимодействия сахароснижающих препаратов с микробиотой кишечника в перспективе может способствовать управлению скорости всасывания препаратов, а также усилению их терапевтических эффектов при минимальном количестве побочных явлений.

## ДОПОЛНИТЕЛЬНАЯ ИНФОРМАЦИЯ

Источники финансирования. Работа выполнена по инициативе авторов без привлечения финансирования.

Конфликт интересов. Авторы декларируют отсутствие явных и потенциальных конфликтов интересов, связанных с содержанием настоящей статьи.

Участие авторов. Все авторы одобрили финальную версию статьи перед публикацией, выразили согласие нести ответственность за все аспекты работы, подразумевающую надлежащее изучение и решение вопросов, связанных с точностью или добросовестностью любой части работы».
